# The prevalence of violence and its association with mental health among the Iranian population in one year after the outbreak of COVID-19 disease

**DOI:** 10.1186/s12888-022-04444-7

**Published:** 2023-01-13

**Authors:** Koorosh Kamali, Azam Maleki, Seyed Abbas Bagheri Yazdi, Elham Faghihzadeh, Zarrintaj Hoseinzade, Marzieh Hajibabaei, Seyedeh Elham Sharafi, Ahmad Ali Noorbala

**Affiliations:** 1 Social Determinants of Health Research Center, Zanjan University of Medical Sciences, Zanjan, Iran; 2 Department of Mental Health, Ministry of Health and Medical Education of Iran, Tehran, Iran; 3 Department of Epidemiology and Biostatistics, School of Medicine, Zanjan University of Medical Sciences, Zanjan, Iran; 4 Psychosomatic Medicine Research Center, Tehran University of Medical Sciences, Tehran, Iran; 5 Department of Psychiatry, Psychiatry and Psychology Research Center, Tehran University of Medical Sciences, Roozbeh Hospital, South Kargar AV., Tehran, 13185/1741 Iran

**Keywords:** COVID-19, Violence, Mental health, Iran

## Abstract

**Background:**

COVID-19 spread between and across nearly every country, with considerable negative health consequences. The current study aimed to determine the prevalence of violence and its association with mental health among Iranians older than 15 years in 2020.

**Methods:**

Data was collected through National Mental Health Survey on 24,584 Iranians older than 15 years in 2020. were analyzed to determine the prevalence of violence and its association with mental health. Multi-stage sampling method was used, and data on demographic characteristics and domestic-social violence and mental health (GHQ-28) were collected. Data analysis was administered using descriptive statistics and a chi-square test at a 95% level.

**Results:**

The mean age of participants was 44.18 ± 16.4 years. The overall prevalence of domestic and social violence was 11.4% and 5.5%, respectively. Verbal violence was the most common type; with 61.8% and 66.8% for domestic and social violence, respectively. A suspected case of mental disorder, female gender, being younger than 25 years, living apart together, unemployment, low education, and history of COVID-19 infection presented a significant association with domestic and social violence (*p* > 0.05).

**Conclusion:**

In comparison to the previous study in 2015, the prevalence of violence has increased. Therefore, domestic and social violence are the social concerns of Iranian society, indicating the necessity of appropriate interventions, particularly for those suspected of mental disorders and young women with low education levels.

## Background

Violence refers to a wide range of behaviors, ranging from humiliation, threats, swearing, and assault to property damage and murder [1]. Violence against women (VAW) violates women's human rights with a severe impact on victims, families, and society [2]. Domestic violence is one of the most common forms of violence against women worldwide and includes physical, financial, and sexual violence as well as emotional and psychological abuse [3].

Violence against women in one study conducted by Ahmadi et al. 2007 was 35.7% and VAW in women with mental health disorders was 3.5 times more than the healthy people [4]. A systematic review of the prevalence of domestic violence against women reported a rate of 48.87% in 2015 [5]). Also, Haj Ansari et al. (2017) reported a prevalence of 66% [6]. A National Study on Mental Health, conducted by Noorbala et al. (2015), reported a prevalence of 23.08% and 8.7% for domestic and social violence, respectively [7], similar to other developing countries [8]).

Until the 2000s, domestic violence was mainly a gender-based issue (i.e., only against women); however, violence against men is also an important issue [9]. Several factors have been identified as potential causes of domestic violence, including economic challenges, sexual problems, alcohol and drug abuse, conflict over parenting, history of child abuse, divorce, and forced marriage [3, 10, 11]. Despite, legalizing the gender change process in Iran, transsexuals still have a high level of discrimination, violence, and suicidal behavior. It shows that these people are exposed to all kinds of threats without sufficient support from their families and society [12]. The experience of sexual violence among Iranian prostitutes indicates an increase in the prevalence of lifelong sexual violence. Also, having a history of drug use, engaging in anal sex, engaging in group sex, having a high number of partners, recent unstable housing, and being incarcerated have increased the likelihood of experiencing sexual violence [13]. Examining the challenges and facilitating factors of creating an interpersonal violence registration system from the perspective of stakeholders shows that the most important challenges include insufficient reporting of victims due to financial problems, as well as psychosocial barriers and structural barriers such as organizational barriers and methodological challenges [14].

Low mental health is also a major contributor to violence; women who report higher levels of domestic violence often suffer from higher levels of psychological distress, anxiety, depression, and loss of emotional/behavioral control than their counterparts [11]. COVID-19 spread between and across nearly every country, with considerable negative health consequences. The World Health Organization (WHO) warned its members that quarantine and social distancing could increase VAW. Regarding the economic and social consequences of COVID-19, the occurrence of VAW is potentially higher than in previous catastrophic events [15]. Yari et al. (2020) reported that during the early COVID-19 outbreak, the prevalence of domestic violence against Iranian women aged 19 to 65 years old was moderate by 57.2% and severe by 26.1% [3]. In addition, the prevalence of violence increased from 54.2%, before the outbreak, to 65.4% [16], indicating the potential role of COVID-19 consequences in domestic violence.

Evidence indicates an increased prevalence of social violence during the COVID-19 outbreak, while street quarrels considerably declined following imposed restrictions [17, 18]. As several factors contribute to violence occurrence and the COVID-19 outbreak began more than a year ago, which is relatively enough to study its social, economic, and public health consequences, it seems that investigating the prevalence of violence and factors contributing to its occurrence in Iran would provide valuable information. Hence, the current study aimed to investigate the prevalence of violence and its association with mental health among the Iranian population one year after the outbreak of COVID-19 disease.

## Methods

Data collected through National Mental Health Survey on 24,584 Iranians older than 15 years from January to February 2020 were analyzed to determine the prevalence of violence and its association with mental health.

## Participants

Inclusion criteria consisted of an Iranian population upper than 15 years old, fluency in the Persian language, and volunteering to participate in the study. All individuals living in a house were considered as the study population. Noteworthy, commercial centers, public organizations (e.g., schools and hospitals), guests, or non-Iranians, and incomplete questionnaires were excluded.

In the current study violence data was derived from the National Mental Health Survey [19]. Assuming the prevalence of psychiatric disorders at 30% (*P* = 0.3), the first type error at 0.05, the accepted error at 0.04, and the effect of cluster sampling equal to 1.6, the sample size for each province was calculated as 825 individuals. Considering 31 provinces, the total sample size was 25,575 individuals.

## Sampling method

**Fig. 1:**
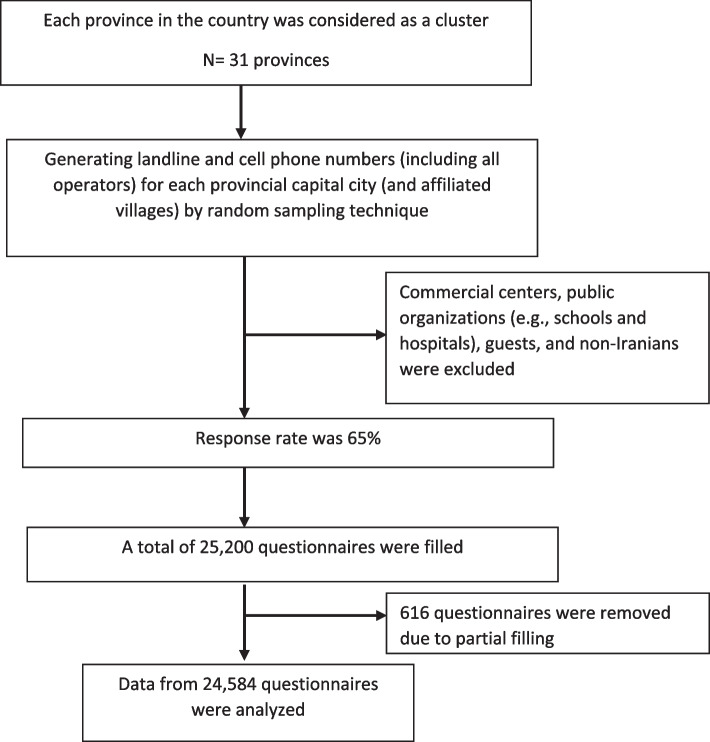
The process of sampling

Multi-stage sampling was used in this study regarding the high penetration of telephones in the country and the lack of a standard protocol to perform surveys during the COVID-19 pandemic. Initially, each province was considered as a cluster (*n* = 31 provinces); then, the sample size of each province was determined based on the statistical population. Afterward, a series of phone numbers, either landline or cellphone, were selected using predefined codes of each province capital by random sampling technique. Nearly two-thirds of subjects were selected using the mobile phone number, and the rest using the landline. After generating landline and cell phone numbers (including all operators) for each provincial capital city (and affiliated villages), the interviewers randomly completed the questionnaires by observing the age and gender ratios. About two-thirds of the samples were assigned to cell phones, and the others were assigned to landlines. Most of the telephone interviews were conducted in Farsi; however, Kurdi and Azari were also used in some provinces. Data supervision and control were performed in parallel. The overall response rate was 65%( Fig. 1).

### Data Collection

Data on demographic information, domestic-social violence, and mental health (GHQ-28) were collected using telephone interviews. The demographic questionnaire contained information on age, gender, marital status, employment, residence area, COVID-19 infection (the interviewee and the family member), infection duration (if infected with COVID-19), and severity of COVID-19 infections.

### Violence Questions

We used the self-designed questionnaire to assess violence that has been used in a previous national study in 2015 [7]. This section of the questionnaire was measured and standardized by a pilot study. The validity and applicability of the questionnaire were evaluated likewise. To evaluate the reliability of the questionnaire, the test–retest method (one week after the initial implementation) was used on 250 people. The Spearman correlation coefficient was 0.8966. The validity of the content of the questions was evaluated by a group of experts in behavioral sciences, statistics, and epidemiologists.

The items were to evaluate the level of exposure to domestic and social violence during the past month. Each dimension of domestic and social violence contains two areas: physical and verbal. The formerly included consequences of physical violence (bruising, rupture, bleeding, fractures, and dislocation) and physical violence with no consequence (Pushing, slapping, and kicking). The latter contained abusive language. The frequency of domestic and/or social violence was evaluated using yes, no, or do not know items; while, the type of violence was evaluated using yes or no items.

### General Health Questionnaire-28 items (GHQ-28)

The GHQ-28 was used to screen the mental health of participants. This questionnaire, initially developed by Goldberg and Hillier (1979), contains four subscales of Somatic symptoms (items 1–7), anxiety/insomnia (items 8–14), social function (items 15–21), and depression symptoms [20–26]. The items are scored on a four-point Likert scale, ranging from never (one) to much better than usual (four). Positive items are reverse-scored. The traditional method was used to calculate the total score of GHQ; items were scored as 1–1-0–0, and the total score ranged from zero to 28. A total score higher than six and a score higher than two for each subscale indicate distress symptoms. Noorbala et al. (2008) confirmed the validity and reliability of the Persian version of GHQ [27].

### Data analysis

Description of data was administered using descriptive statistics (frequency, percentage, mean, and standard deviation). To calculate the national prevalence of violence, weighting was performed according to the population of each province. A chi-square test was used to study the relationship between all demographic variables and violence through the SPSS 16 software (SPSS Inc, Chicago, USA). Statistical significance was considered at the *p*-value < 0.05.

## Results

A total of 25,200 questionnaires were filled, of which 616 questionnaires were removed due to partial filling. Hence, data from 24,584 questionnaires were analyzed. The mean age of participants was 44.18 ± 16.47 years. Most participants were living in urban areas (76.25%), male (50.07%), aged 25 to 44 years (37.61%), educated up to Diploma and Bachelorette (26.38%), employed (40.32%), and married (73.36%). Concerning the type of residence area, most participants owned a house (79.5%) (Table 1).

**Table 1:** Distribution of demographic status of participants

Demographic factors	Sample size	
	N	%
Gender		
Male	12,309	50.07%
Female	12,275	49.93%
Residency		
Urban	17,860	72.65%
Rural	6724	27.35%
Age groups		
15–24	3373	13.73%
25–44	9242	37.61%
45–64	8528	34.71%
≥ 65	3429	13.95%
Marriage status		
Married	17,948	73.36%
Widowed	1306	5.34%
Divorced	313	1.28%
Single	4837	19.77%
Separated	61	0.25%
Education		
Illiterate and Read &Wright	4380	17.90%
Elementary & secondary	5644	23.07%
Diploma	6172	25.22%
Above diploma &Bachelor	6454	26.38%
Master and above	1818	7.43%
Job status		
Employed	9860	40.32%
Unemployed	1553	6.35%
Student	2071	8.47%
Housewife	7963	32.56%
Retired & Pensioner	2794	11.43%
Unable to work	214	0.88%

In addition, 14.1% of participants had a history of COVID-19 infection, mostly minor infection (51.7%). The hospitalization period due to COVID-19 ranged from one to three months (52.1%). The rate of COVID-19 infection among family members and other relatives was 32.3%; while 13.2% reported losing a loved one due to COVID-19 infection (Table 2).

**Table 2:** Distribution of Covid-19 infection in their family

Variable	Sample size	
	N	%
Infected by Covid-19		
Yes	3624	14.7
No	20,958	85.3
Intensity of the infection		
Mild	1868	51.7
Moderate	1099	30.4
Severe	646	17.9
Time passed since the infection (month)		
< 1	128	3.6
1–3	1863	52.1
4–6	866	24.2
7–9	307	8.6
10–12	409	11.4
Family members and other relatives infected by Covid-19 disease		
Yes	7928	32.3
No	16,643	67.7
Losing a loved one due to Covid-19 infection		
Yes	1050	13.2
No	6884	86.8

### Prevalence of violence and its association with mental health

The overall prevalence of domestic and social violence in those older than 15 years was 11.4% and 5.5%, respectively. Verbal violence was the most common type; with a prevalence of 61.8% and 66.8%, respectively, for domestic and social violence. Nearly 29.7% of those older than 15 years were suspected of mental disorders. Violence was significantly associated with mental health; so, the prevalence of domestic (21.6%) and social violence (9.6%) in cases suspected of mental disorder was considerably higher than in healthy subjects (*p* = 0.001). In addition, they experienced the highest rate of domestic violence (Table 3).

**Table 3:** The Prevalence of domestic and social violence and its relationship with mental health among participants

Violence		Yes		No		I don't know		Mental health status				
		Frequency	Percentage	Frequency	Percentage	Frequency	Percentage	GHQ ≥ 6		GHQ < 6		*P* value
								Yes	No	Yes	No	
Total score	Domestic	2808	11.4	21,689	88.3	79	0.3	21.60	78.40	7.60	92.40	< 0.001
Total score	Social	1350	5.5	23,148	94.2	65	0.3	9.60	90.40	4.10	95.90	< 0.001
Type of domestic violence	physical violence with consequence	49	1.9	2475	98.1	-		2.70	97.30	1.00	99	0.002
	physical violence with no consequence	289	11.5	2235	88.5	-		13.70	86.30	8.70	91.30	< 0.001
	Verbal	1561	61.8	963	38.2	-		63.20	36.80	59.80	40.20	0.086
Type of social violence	physical violence with consequence	61	5	1157	95	-		7.40	92.60	2.70	97.30	< 0.001
	physical violence with no consequence	149	12.2	1069	87.8	-		14.70	85.30	10.20	89.80	0.017
	physical violence with consequence	814	66.8	404	33.2	-		65.30	34.70	67.70	32.30	0.388

### Social and economic factors related to domestic violence

The highest percentage of domestic violence was reported in women (12.1%), those living in rural areas (11.6%), aged 15 to 24 years (13.5%), those living apart together (19.6%), primary education (up to middle school degree; 12.7%), and unemployed subjects (16.4%). The association between demographic characteristics and domestic-social violence is provided in Table 3. According to the results, variables of gender, age, marital status, employment, and education were significantly associated with domestic violence. Meanwhile, the residence area presented a reverse association (Table 3).

### Social and economic factors associated with social violence

The highest percentage of social violence was reported in men (8.6%), those living in urban areas (5.9%), aged 15 to 24 years old (8.1%), divorced (11.2%), with a university degree (6.9%), and employed (8.6%). Variables of gender, age, residence area, marital status, employment, and education showed a significant association with social violence (Table 4).

**Table 4:** Distribution of domestic and social violence in term of economic and social characteristics of participants

	Violence					
Variables	Domestic Violence			Social Violence		
	Frequency	(%)	*P* value	Frequency	(%)	*P* value*
Gender						
male	1324	10.8	> 0.003	1055	8.6	> 0.001
female	1484	12.1		295	2.4	
Residency						
Urban	2026	11.3	0.424	1045	5.9	> 0.001
Rural	782	11.6		305	4.5	
Age groups						
15–24	455	13.5	> 0.001	273	8.1	> 0.001
25–44	1198	13		659	7.1	
45–64	872	1.2		354	4.2	
≥ 65	282	8.2		63	1.8	
Marriage status						
Married	2040	11.4	> 0.001	839	4.7	> 0.001
Widowed	126	9.6		26	2	
Divorced	53	16.9		35	11.2	
Single	12	19.7		5	8.2	
Separated	570	11.8		441	9.1	
Education						
Illiterate and	450	10.3	> 0.001	94	2.1	> 0.001
Read &Wright	719	12.7		309	55	
Elementary & secondary	742	12		396	6.4	
Diploma	715	11.1		416	6.4	
Above diploma &	174	9.6		126	6.9	
Job status						
Bachelor	1066	10.8	> 0.001	850	8.6	> 0.001
Master and above	254	16.4		132	8.5	
Employed	256	12.4		135	6.5	
Unemployed	983	12.3		142	1.8	
Student	32	15		8	3.7	
Housewife	206	7.4		78	2.8	

^*^chi-square test

### Association between COVID-19 infection and violence

A significant association was found between COVID-19 infection and social violence; so that violence was more prevalent in subjects infected with COVID-19 (15.6%), those with a history of COVID-19 in family members (14%), and losing a loved one due to COVID-19 (15.7%) than those with no history of COVID-19 infection. Nevertheless, social violence was only significantly associated with the status of COVID-19 infection (Table 5).

**Table 5:** Distribution of domestic and social violence in term of affected by Covid-19 Disease

	Domestic Violence			Social Violence		
	Frequency	(%)	*P* value	Frequency	(%)	*P* value
Covid-19 infection						
yes	565	15.6	> 0.001	245	6.8	> 0.001
no	2243	10.7		1105	5.3	
A history of Covid-19 in family members						
yes	1107	14	> 0.001	450	5.7	0.280
no	1698	10.2		898	5.4	
Losing a loved one due to Covid-19						
yes	165	15.7	0.035	71	6.8	0.101
no	943	13.7		381	5.5	

## Discussion

In this study, data collected through National Mental Health Survey were analyzed to investigate the prevalence of violence and its association with mental health. Domestic and social violence prevalence among those older than 15 years was obtained as 11.4% and 5.5% in one month. With a prevalence of 61.8% and 66.8% for domestic and social violence, respectively, verbal violence was the most common type. Female gender, being younger than 25 years old, living apart together, unemployment, and low education level were significantly associated with domestic and social violence. The National Study of Mental Health, conducted by Noorbala et al. (2015), reported a one-year prevalence of 23.08% for domestic violence, 23.65%, and 22.52% for males and females, respectively. A prevalence of 8.7% is reported for social violence; 12 and 4.14% for males and females, respectively [7].

The observed reduced prevalence of violence, as compared to 2015, should not be interpreted as a declining trend because of the increased number of contacts with social emergency services since the onset of the COVID-19 outbreak and the negative consequences of the pandemic, including social and economic, indicate the increased occurrence of violence [28]. Noteworthy, their study investigated the point prevalence (one month) of violence, while our study reported a one-year prevalence. Hence, the simple comparison of these two studies indicates a declining trend.

Although the government has devised various solutions over many years to deal with and reduce the problem of violence in different social groups, it is not enough and this problem is still prevalent, especially in the villages. Women's ignorance of their rights, which are stated in the constitution and civil laws, is the main factor of mental and physical vulnerability of people [20]. Although domestic violence is a global phenomenon, it can have different definitions and rates of occurrence depending on the culture in which it occurs. In addition, unlike in Western countries where it is relatively easy for researchers to obtain and access information on various aspects of family violence, it is a big challenge in some countries, including Iran, due to inaccurate information [14]. It is necessary for the criminal justice system at the level of legislation and implementation, using the experiences of other countries, to expand appropriate measures to ensure the right security of people against crime and victimization and to protect their right to aggression.

Several studies investigated domestic and social violence in Iran before and after the COVID-19 outbreak. A meta-analysis of studies performed before the COVID-19 outbreak reported a prevalence of 52% for psychological violence against women, 37% for physical violence, and 34% for social violence. In addition, the highest rate of violence against women is reported for those aged 20 to 30 years old (48.5%), and domestic violence against housewives is reported as 65.3% [21] which both domestic and social violence is higher than that of the present study. The observed difference can be attributed to the type of studies. Studies on VAW reported a prevalence of 2.3 to 73.78% for different developing countries. Physical and emotional-psychological violence prevalence ranges from 61.6 to 11.54% and 7.8 to 84.3%, respectively. Prevalence of sexual, economic, and verbal violence is reported as 0.8–58.8%, 13.7–43.7%, and 33.21–86.1%, respectively. The most common causes of VAW are structural, including early marriage and husband addiction, either drugs or alcohol [8].

Few studies have investigated the prevalence of violence since the COVID-19 outbreak in the general population of Iran. Yari et al. (2020) reported that during the early COVID-19 outbreak, the prevalence of domestic violence against Iranian women aged 19 to 65 years old was moderate by 57.2% and severe by 26.1%. They reported higher rates of psychological and sexual violence. The prevalence of domestic violence was higher among women younger than 25 years and illiterate women [3]. A study intended to compare the prevalence of domestic violence among women aged 18 to 60 years old before and after the COVID-19 outbreak reported an increasing trend (i.e., from 54.2 to 65.4%). Furthermore, 25.5% of women had experienced domestic violence for the first time in their life after the COVID-19 outbreak, with a higher rate of psychological violence (14.7%) in comparison to physical (7%) and sexual violence (8.4%). The prevalence of domestic violence in women older than 50 years, housewives, illiterate women, and low-income families was significantly higher than in other groups [16] Regardless of the administered tool to measure domestic violence in these two studies, it seems that, based on the present study's findings, the prevalence of violence has declined since the early months of the COVID-19 outbreak. However, concerning the variable of age and its association with domestic violence, the findings of this study are not in line with Fereydony's study; while, Yari et al. reported similar results. The observed difference can be attributed to the study population and administered tool. A study conducted in Portugal on cases older than 16 years old reported similar results; an overall prevalence of 13.7% for violence. In contrast to the findings of this study, the Portugal study reported higher rates of psychological violence (13%) in comparison to sexual and physical violence [22].

Women and children are among the most vulnerable populations. Violence against women and children has a long history worldwide, regardless of social, economic, and cultural class or ethnicity, or race [23]). Since the onset of the COVID-19 outbreak, stay at home policy has been emphasized by several countries, which according to the evidence, is accompanied by an increased occurrence of violence worldwide. For instance, a review study reported that its prevalence has increased by 48% in the USA in comparison to before the COVID-19 outbreak [24]. Therefore, countries should be aware that encouraging people to respect quarantine to cope with SARS-CoV-2 infection has turned into a paradox in terms of domestic violence. Women who have to spend time at home may experience social isolation, leading to the inability to seek help [25]. Meanwhile, the WHO warned its members that quarantine, isolation, and social distancing could increase violence against women [2]. Increased occurrence of VAW, particularly among younger women, indicates a severe barrier to improving gender inequalities. In other words, these findings should sound the alarm regarding the need for planning and introducing appropriate and in-time interventions to address its consequences.

Violence is often associated with increased psychological disorders [26]. Also, prolonged exposure to stressors in women with a history of domestic violence is associated with an increased prevalence of post-traumatic stress disorder and depression. Meanwhile, increased resilience is reported as an adjusting factor in coping with these women's psychological disorders [29]. According to the findings, the prevalence of domestic and social violence in cases suspected of mental disorders was significantly higher than in healthy subjects.

In a study by Mengo et al., 68.5% of women suffering from mental disorders experienced various levels of physical violence, and 71.4% reported sexual violence, which is higher than the rates of the present study [30]. The observed difference can be attributed to the impact of cultural factors and how each individual interprets violence in various communities. In addition, it should be noted several cases of violence goes unreported.

Many factors are reported to affect the increased occurrence of violence following catastrophes. For instance, such events may cause reduced marital satisfaction and increased aggressive behaviors, leading to declined intimacy. In addition, stressful events following a catastrophe result in economic disruption or uncertainty, or psychological disorder, leading to an increased prevalence of aggressive behaviors of the partner. Furthermore, access to external support, including family members and friends, or professional services, which are highly useful for victim women, is often declined [31, 32]. Therefore, measures are needed to address the negative consequences of quarantine and stay-at-home policies.

Few studies investigated the prevalence of social violence during the COVID-19 outbreak in Iran. On the other hand, in some countries, including Australia, a considerable decline in social violence, such as sexual abuse and quarrel, is reported following the stay-at-home policy compared to the previous year [12]. Meanwhile, the rate of domestic violence did not change. Similar results are reported in Sweden and USA [18, 33]. Nevertheless, a study reported a slight increase in vehicle theft during the COVID-19 outbreak [34]. In the same vein, the findings of this study also indicated a lower prevalence of social violence than domestic violence. There was a significant association between COVID-19 infection and domestic and social violence. COVID-19 infection and its social stigma may pave the way for domestic and social violence [35], indicating the necessity of increased attention from planners and health policymakers.

### Strengths

Following a population-based design and selecting a high number of subjects using a random sampling technique are among the strengths of this study. In addition, the prevalence of violence in both sexes is considered. Last but not least, participants are selected from all provinces of the country.

### Limitations

In this study, data are collected using the self-report method by telephone interview. Also, only physical and verbal dimensions of violence are investigated. Regarding the importance of other dimensions of violence, including physical and mental, and its severe consequences, particularly during the COVID-19 outbreak, caution should be taken when generalizing the results.

## Conclusion

It can be concluded that the prevalence of social violence is lower than domestic violence but it seems that the prevalence of violence has increased in Iran since 2014. Therefore, domestic violence is a social emergency in the Iranian community still. This study only investigated physical and verbal violence, and other types of violence should also be considered to extend our knowledge, particularly in areas with high rates of violence.

## Implications of the study

The findings of this study, in addition to obtaining basic information about the prevalence of domestic and social violence and its relationship with the mental health of the Iranian population one year after the outbreak of the COVID-19 epidemic, have also revealed some related factors. It can be used to design appropriate interventions, particularly among those suspected of psychological disorders, women, living apart together, unemployed, younger than 25 years, and with low education.

## Data Availability

The dataset used in the present study is available from the corresponding author upon reasonable request.

## Abbreviations

VAW: Violence against women
USA: The United States of America
COVID-19: Coronavirus disease 2019
GHQ: General Health Questionnaire
WHO: World Health Organization
